# Multiple Antibiotic Resistance of *Vibrio cholerae* Serogroup O139 in China from 1993 to 2009

**DOI:** 10.1371/journal.pone.0038633

**Published:** 2012-06-11

**Authors:** Li Yu, Yanyan Zhou, Ruibai Wang, Jing Lou, Lijuan Zhang, Jie Li, Zhenqiang Bi, Biao Kan

**Affiliations:** 1 State Key Laboratory for Infectious Disease Prevention and Control, Department of Diarrheal Diseases, National Institute for Communicable Disease Control and Prevention, Chinese Center for Disease Control and Prevention, Beijing, People’s Republic of China; 2 Shandong Provincial Key Laboratory of Infectious Disease Control and Prevention, Shandong Center for Disease Control and Prevention, Jinan, China; Johns Hopkins Bloomberg School of Public Health, United States of America

## Abstract

Regarded as an emerging diarrheal micropathogen, *Vibrio cholerae* serogroup O139 was first identified in 1992 and has become an important cause of cholera epidemics over the last two decades. O139 strains have been continually isolated since O139 cholera appeared in China in 1993, from sporadic cases and dispersed foodborne outbreaks, which are the common epidemic types of O139 cholera in China. Antibiotic resistance profiles of these epidemic strains are required for development of clinical treatments, epidemiological studies and disease control. In this study, a comprehensive investigation of the antibiotic resistance of *V. cholerae* O139 strains isolated in China from 1993 to 2009 was conducted. The initial O139 isolates were resistant to streptomycin, trimethoprim-sulfamethoxazole and polymyxin B only, while multidrug resistance increased suddenly and became common in strains isolated after 1998. Different resistance profiles were observed in the isolates from different years. In contrast, most *V. cholerae* O1 strains isolated in the same period were much less resistant to these antibiotics and no obvious multidrug resistance patterns were detected. Most of the non-toxigenic strains isolated from the environment and seafood were resistant to four antibiotics or fewer, although a few multidrug resistant strains were also identified. These toxigenic O139 strains exhibited a high prevalence of the class I integron and the SXT element, which were rare in the non-toxigenic strains. Molecular subtyping of O139 strains showed highly diverse pulsed-field gel electrophoresis patterns, which may correspond to the epidemic state of sporadic cases and small-scale outbreaks and complex resistance patterns. Severe multidrug resistance, even resistance transfers based on mobile antibiotic resistance elements, increases the probability of O139 cholera as a threat to public health. Therefore, continual epidemiological and antibiotic sensitivity surveillance should focus on the occurrence of multidrug resistance and frequent microbial population shifts in O139 strains.

## Introduction

Cholera is a major public health problem in developing countries. The cholera toxin of the *Vibrio cholerae* strains, encoded by *ctxAB* in the lysogenic bacteriophage CTXΦ, is the major virulence factor responsible for diarrhea. Historically, only the *V. cholerae* O1 serogroup has been recognized to be associated with epidemics in the sixth and seventh cholera pandemics. The *V. cholerae* O139 strains emerged as a new serogroup in southern India and Bangladesh in late 1992 and rapidly spread to all cholera-endemic areas in India and neighboring countries [1], [2]. A new clone of the *V. cholerae* O1 El Tor biotype replaced the O139 serogroup as the dominant serogroup causing cholera in 1994, when few O139 cholera cases were reported [3]. During late 1995 and 1996, both *V. cholerae* O1 and O139 cases were detected in Bangladesh and since 1996, cholera in Bangladesh has been caused predominantly by *V. cholerae* O1 El Tor [4]. An extensive outbreak of cholera caused predominantly by *V. cholerae* O139 recurred in some regions of Bangladesh in 2002 [4]. A similar cholera outbreak also appeared in some parts of India during August, 1996 and September, 1997 and during September and October of 1998, when O139 cholera cases were predominantly observed [5], [6], [7], [8], [9]. The first outbreak caused by serogroup O139 strains in China occurred in Xinjiang in 1993, when approximately 200 cases were reported [10]. Unlike the explosive epidemics in Bangladesh and India, the O139 cholera outbreaks spanning different regions in China were rare. Limited foodborne outbreaks associated with food poisoning and sporadic cases in various regions, especially in southeastern China, were frequently reported and have been expanding since 1998. To date, serogroup O139 continues to be confined to Southeast Asia, while the threat of global expansion is ever-present.

The primary treatment for cholera is rehydration with oral or intravenous fluids. Antibiotic therapy can shorten the duration and severity of diarrhea but also aggravates the appearance of antibiotic resistance [11]. Analysis of O139 isolates revealed interesting patterns of resistance to various common antibiotics [9], [12]. In contrast to *V. cholerae* O1, the initial O139 isolates were resistant to streptomycin and trimethoprim-sulfamethoxazole [2] due to the presence of a 62 kb integrating conjugative element, termed SXT [13]. *V. cholerae* O139 isolates have been frequently reported to be resistant to various commonly used antibiotics, such as ampicillin, tetracycline, chloramphenicol, nalidixic acid, nitrofurantoin and even to ciprofloxacin [12], [14], [15], [16], [17]. According to a previous report, almost all 352 *V. cholerae* O139 strains isolated from hospitalized clinical cases and environmental sources from 1997 to 1998 were reported to be resistant to ampicillin, furazolidone and neomycin [18]. Ofloxacin-resistant O139 strains were reported in Hong Kong in 1998 [19]. Furthermore, the multidrug resistant (MDR) *V. cholerae* has received increasing worldwide attention especially in the epidemic regions [4].

The mechanisms of antimicrobial resistance are associated with intrinsic resistance, acquired genetic determinants of resistance and mutations. The spread of MDR *V. cholerae* O139 strains has been attributed to the mobilization of drug resistance genetic elements and MDR in *V. cholerae* has traditionally been described upon acquisition of R plasmids [17], [20], [21]. Other genetic elements, including the class I integron and the SXT element, have been reported to be closely associated with the spread of genetic elements by mediating antibiotic resistance in *V. cholerae* [22], [23].

Antibiotic resistance in *V. cholerae* O139 strains in China has not been comprehensively reported previously. In this study, antimicrobial susceptibility to 16 different antibiotics was investigated among *V. cholerae* O139 strains isolated in China from 1993 to 2009, especially in comparison with the contemporaneous serogroup O1 strains. Mobile genetic elements associated with antibiotic resistance were also characterized to further elucidate the mechanisms of antibiotic resistance in serogroup O139 strains.

## Results

### Antibiotic Susceptibility of Toxigenic and Non-toxigenic O139 Strains

PCR analysis of the cholera toxin revealed that 85.3% (290/340) of the *V. cholerae* O139 strains in this study were positive for the 749 bp amplicon. Most toxigenic O139 strains were isolated from patient samples, while non-toxigenic strains were almost isolated from environmental water and animals. In order to characterize the variance in antibiotic resistance between toxigenic strains and non-toxigenic strains, the antibiotic susceptibility patterns of 340 *V. cholerae* O139 isolates were analyzed (Table 1). Briefly, high rates of resistance were detected among toxigenic and non-toxigenic O139 strains to erythromycin, streptomycin and polymyxin B (94.1%/98.0%, 97.9%/88.0% and 99.0%/86.0% respectively). Comparative analysis revealed significantly higher rates of resistance to nalidixic acid, trimethoprim-sulfamethoxazole, tetracycline, chloramphenicol, azithromycin and kanamycin among the toxigenic O139 strains compared with the non-toxigenic strains *(P<*0.01, χ^2^ test). A high rate of resistance (>60%) to ampicillin was detected with less variance among both the toxigenic and non-toxigenic O139 strains. Most toxigenic and non-toxigenic strains were susceptible to new generation antibiotics, such as ciprofloxacin and doxycycline. Only nine toxigenic isolates were resistant to ciprofloxacin, with a minimum inhibitory concentration (MIC) of 4 to 8 µg/ml. Twenty-two toxigenic and one non-toxigenic isolates were resistant to doxycycline, with MICs ranging from 16 to 32 µg/ml. No isolates were resistant to cefuroxime, cefixime and ceftriaxone and few strains were resistant to cephalothin.

**Table 1:** Antibiotic susceptibility patterns of toxigenic and non-toxigenic *V. cholerae* O139 isolates.

Antibiotic	Breakpoints(µg/ml)	Toxigenic (n=290)		Non-toxigenic (n=50)	
		R(%)	S(%)	R(%)	S(%)
Ampicillin	S<=8 R>=32^a^	211(72.7)	79(27.3)	31(62.0)	19(38.0)
Cephalothin	S<=8 R>=32^b^	46(15.9)	244(84.1)	3(6.0)	47(94.0)
Cefuroxime	S<=8 R>=32^b^	0(0)	290(100.0)	0(0)	50(100.0)
Ceftriaxone	S<=8 R>=64^b^	0(0)	290(100.0)	0(0)	50(100.0)
Cefixime	S<=1 R>=4^b^	0(0)	290(100.0)	0(0)	50(100.0)
Nalidixic acid*	S<=16 R>=32^b^	241(83.1)	49(16.9)	13(26.0)	37(74.0)
Ciprofloxacin	S<=1 R>=4^b^	16(5.5)	274(94.5)	0(0)	50(100.0)
Tetracycline*	S<=4 R>=16^a^	242(83.4)	48(16.6)	8(16.0)	42(84.0)
Doxycycline	S<=4 R>=16^b^	39(13.5)	251(86.5)	1(2.0)	49(98.0)
Chloramphenicol*	S<=8 R>=32^a^	193(66.5)	97(33.5)	5(10.0)	45(90.0)
Erythromycin	S<=.5 R>=8^b^	273(94.1)	17(5.9)	49(98.0)	1(2.0)
Azithromycin*	S<=2 R>=8^b^	146(50.3)	144(49.7)	1(2.0)	49(98.0)
Kanamycin*	S<=16 R>=64^b^	231 (79.6)	59(20.4)	6(12.0)	44(88.0)
Streptomycin	S<=4 R>=16^b^	284(97.9)	6(2.1)	44(88.0)	6(12.0)
Polymyxin B	S<=2 R>=4^b^	287(99.0)	3(1)	43(86.0)	7(14.0)
Trimethoprim-Sulfamethoxazole*	S<=2 R>=4^a^	263(90.7)	27(9.3)	16(32.0)	34(68.0)

a Breakpoints are based on the CLSI standards for *V. cholerae*.

b Other breakpoints refer to the CLSI criteria for *Enterobacteriaceae*.

R: includes intermediate and resistance; S: sensitivity.

A single asterisk (*) indicates that the rates of resistance among the toxigenic O139 strains was significantly different (*P*<0.01, *χ^2^* test) from the non-toxigenic strains.

**Figure 1:**
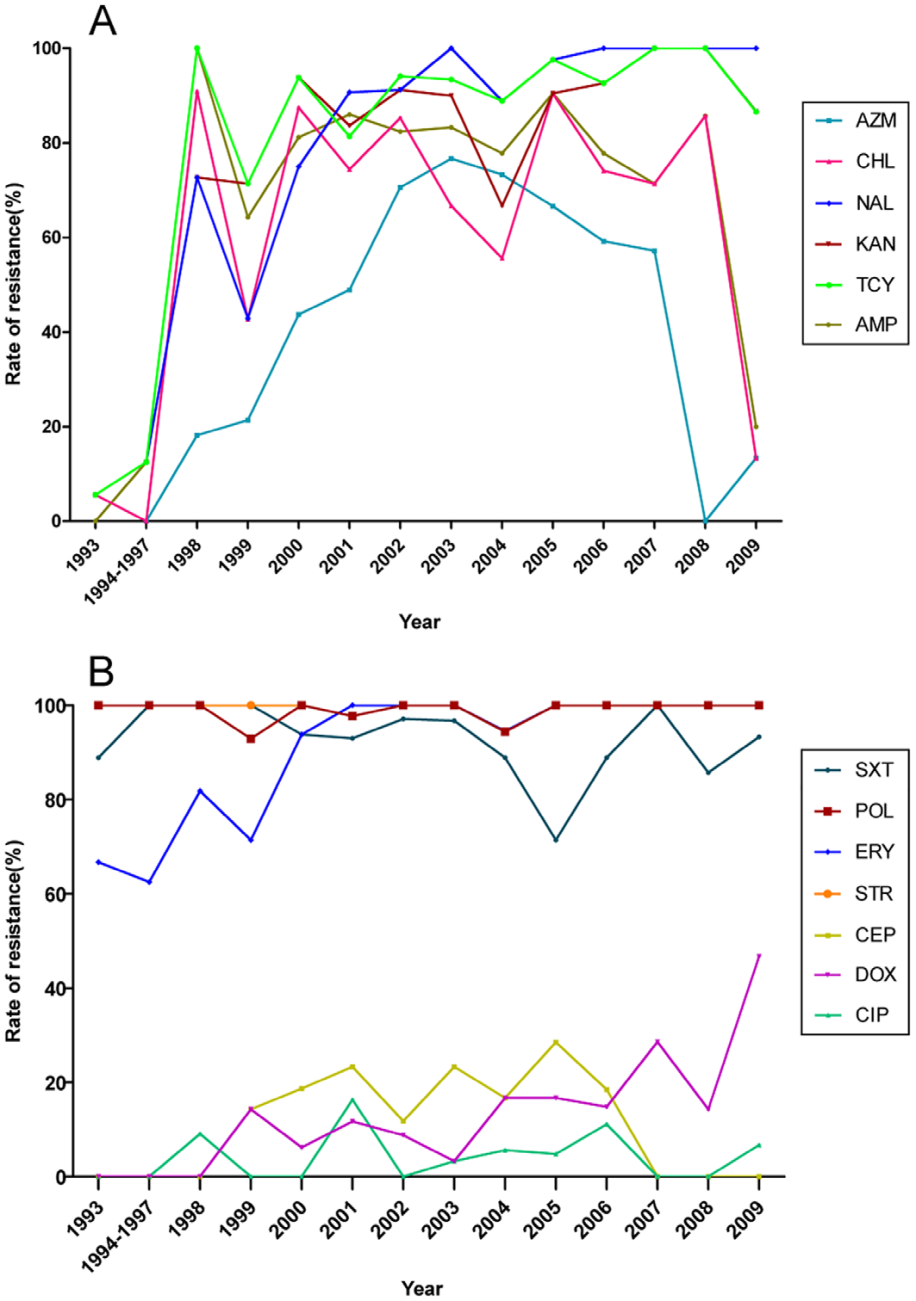
Trends in antibiotic resistance among toxigenic *V. cholerae* O139 strains isolated from 1993 to 2009. Isolates from 1994 to 1997 were merged as one group for their less amounts of strains in each year. A and B: schematic of the dynamics of resistance to antibiotics and cefuroxime, ceftriaxone and cefixime. Non-resistant strains are not listed. AMP: ampicillin, CEP: cephalothin, NAL: nalidixic acid, CIP: ciprofloxacin, CHL: chloramphenicol, TCY: tetracycline, DOX: doxycycline, AZM: azithromycin, KAN: kanamycin, STR: streptomycin, ERY: erythromycin, SXT: trimethoprim-sulfamethoxazole, PB: polymyxin B.

The toxigenic O139 strains isolated from the first outbreak in Xinjiang in 1993 exhibited a higher rate of resistance to streptomycin, trimethoprim-sulfamethoxazole, erythromycin and polymyxin B, which is consistent with previous reports [2]. The rates of resistance to these four antibiotics remained high and stable over the period 1993 to 2009. As shown in Figure 1, most toxigenic O139 strains were susceptible to ampicillin, nalidixic acid, tetracycline, chloramphenicol and kanamycin during the period from 1993 to 1997, while the rates of resistance to these antibiotics increased dramatically, to more than 60% in 1998 and have fluctuated at high levels subsequently. Single-dose azithromycin was used as an effective treatment for severe cholera in adults [21] until the resistant rates increased dramatically and peaked in 2003. Interestingly, the resistance rates to ampicillin, chloramphenicol and azithromycin have decreased significantly in recent years. Most toxigenic O139 strains remain susceptible to cephalothin and ciprofloxacin, while toxigenic O139 strains have exhibited increasing resistance to doxycycline in recent years. The recent alteration in resistance to ampicillin, chloramphenicol, azithromycin and doxycycline may associate with the common usage of these antibiotics in clinical treatments and population shift of the O139 strains with different resistance patterns. In contrast to toxigenic strains, the non-toxigenic O139 strains showed no obvious variance in the resistance to these antibiotics over the period from 1994 to 2009.

### Multidrug Resistance Patterns of Toxigenic and Non-toxigenic O139 Strains

The absence of susceptibility to ampicillin, tetracycline, chloramphenicol, erythromycin, kanamycin, nalidixic acid, streptomycin, trimethoprim-sulfamethoxazole and polymyxin B (AMP-TCY-CHL-ERY-KAN-NAL-STR-SXT-PB) represented the most common pattern of multiple-drug resistance (MDR) and 153 O139 isolates (45.0%) conformed to this pattern (Table S1). Within this pattern group, 151 isolates were positive for cholera toxin, while only two non-toxigenic strains isolated in 2007 exhibited this MDR pattern. The strains exhibiting this pattern of multiple antibiotic resistances first emerged in 1998, and have accounted for approximately half of the strains isolated each year over the period from 1998 to 2008, indicating a predominant dissemination of this MDR pattern. Nineteen toxigenic strains exhibited the second MDR type of ERY-KAN-NAL-STR-SXT-TCY-PB. Only 33 toxigenic strains were resistant to four types of antibiotics or fewer, and 22 of these were isolated before 1998. With the exception of two strains exhibiting the AMP-TCY-CHL-ERY-KAN-NAL-STR-SXT-PB pattern, 74.0% (37/50) of the non-toxigenic strains were resistant to four antibiotics or fewer without obvious temporal distribution. Furthermore, 16 of these non-toxigenic strains were resistant to ampicillin, erythromycin, polymyxin B and streptomycin, thus representing the major MDR pattern for non-toxigenic strains. Only two toxigenic and five non-toxigenic strains were resistant to fewer than two antibiotics and none of the isolates were pan-susceptible to all 16 antibiotics tested. In short, the toxigenic strains exhibited a common pattern of resistance to erythromycin, polymyxin B, streptomycin, trimethoprim-sulfamethoxazole before 1997, with the subsequent emergence of toxigenic strains exhibiting a predominant MDR pattern of AMP-TCY-CHL-ERY-KAN-NAL-STR-SXT-PB after 1998. In contrast, the non-toxigenic strains exhibited less antibiotic resistance than the toxigenic strains with fewer changes in the pattern of resistance over time.

### Comparison of Antibiotic Susceptibility in Toxigenic O1 and O139 Strains Isolated during the Same Period

**Figure 2:**
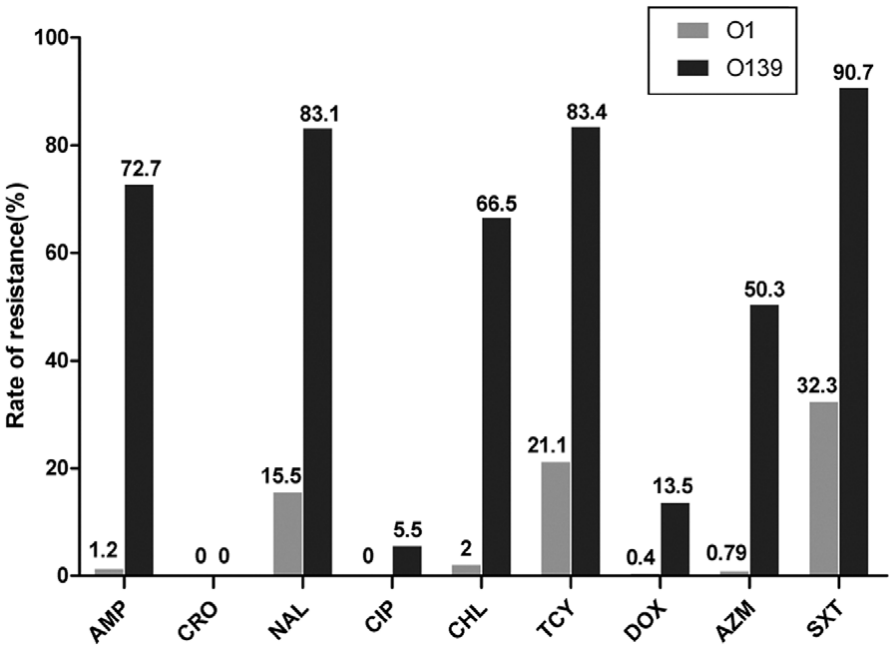
Comparison of antibiotic resistance among toxigenic *V. cholerae* O1 and O139 strains isolated from 1993 to 2009. With the exception of ceftriaxone, O1 strains exhibited significantly lower resistance rates to eight antibiotics compared with O139 strains *(P<*0.01, Wilcoxon rank sum test or χ^2^ test). AMP: ampicillin, CRO: ceftriaxone, NAL: nalidixic acid, CIP: ciprofloxacin, CHL: chloramphenicol, TCY: tetracycline, DOX: doxycycline, AZM: azithromycin, SXT: trimethoprim-sulfamethoxazole.

Antibiotic resistance to nine antibiotics (ampicillin, ceftriaxone, nalidixic acid, ciprofloxacin, chloramphenicol, tetracycline, doxycycline, azithromycin and trimethoprim-sulfamethoxazole) was compared in toxigenic *V. cholerae* O1 (from our database) and O139 strains isolated over the same period from 1993 to 2009. Interestingly, O1 strains exhibited significantly lower rates of resistance to eight antibiotics compared with the O139 strains, with the highest rate of resistance (32.3%) detected to trimethoprim-sulfamethoxazole (Fig. 2). Few toxigenic O1 strains were resistant to ampicillin, chloramphenicol and azithromycin, whereas resistance to these antibiotics was common for toxigenic O139 strains. Of the toxigenic O1 strains, 56.0% were pan-susceptible to all 9 antibiotics tested, while this characteristic was not observed in any of the O139 strains. Of the O1 strains, 22.8% were resistant to more than one antibiotic, and only three O1 strains exhibited the most comprehensive multidrug resistance pattern of ampicillin, nalidixic acid chloramphenicol, tetracycline and trimethoprim-sulfamethoxazole, which was commonly observed among the O139 strains.

### Pulsed-field Gel Electrophoresis Patterns of *V. cholerae* O139 Isolates

**Figure 3:**
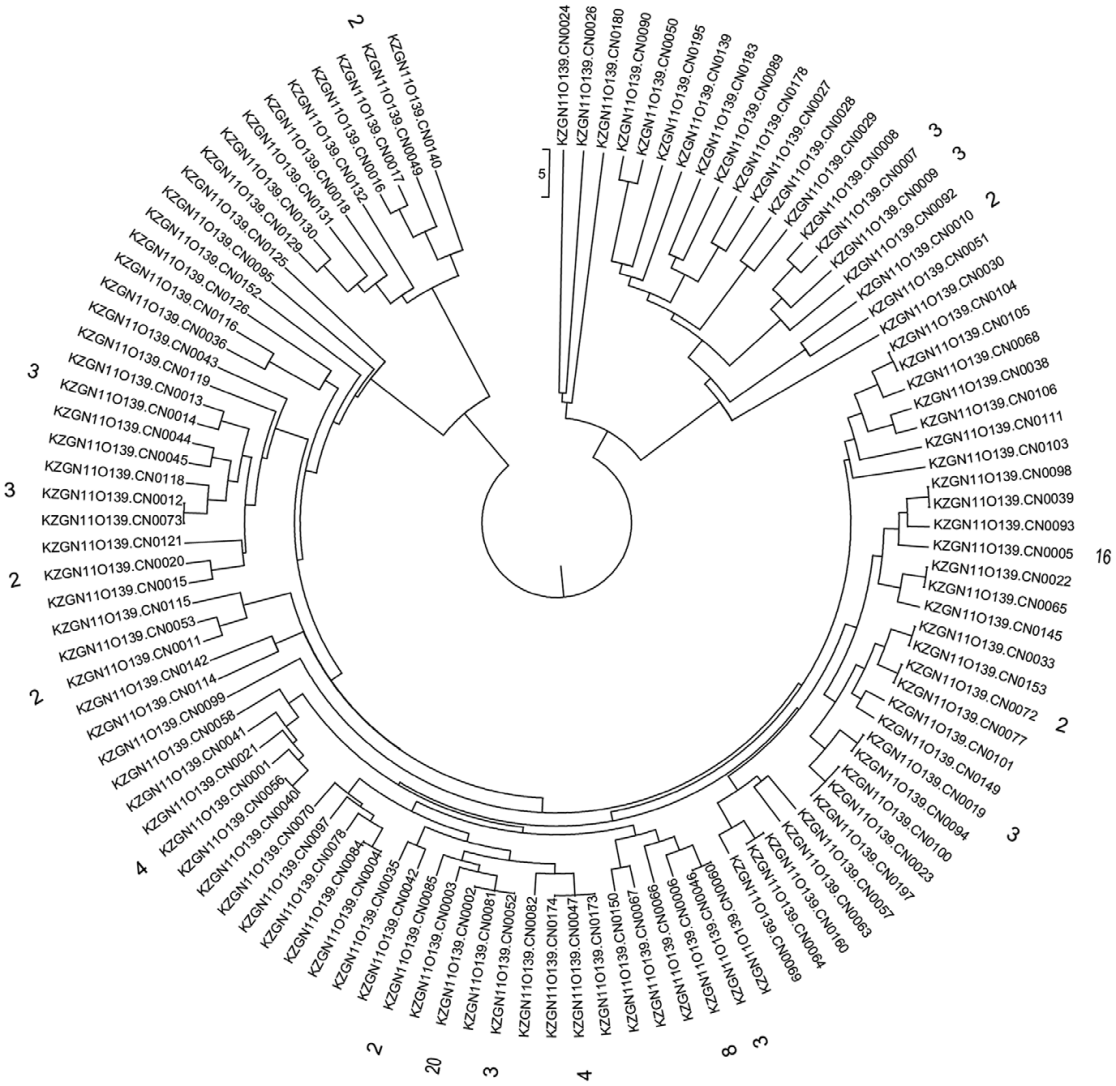
PFGE profiles (*Not* I) obtained from 177 *V. cholerae* O139 strains isolated in China from 1993 to 2009. PFGE patterns (101) are clustered, of which PFGE pattern with 1 strain are not listed.

Pulsed-field gel electrophoresis (PFGE) was used for subtyping of strains to investigate the clonality or genetic variation of serogroup O139 isolates. PFGE of *Not* I digested chromosomal DNA from a subset of 177 isolates generated 101 PFGE patterns (Fig. 3). The most common PFGE pattern, CN0002, was detected for 20 toxigenic isolates, with 14 of these exhibiting the predominant MDR pattern of AMP-TCY-CHL-ERY-KAN-NAL-STR-SXT-PB. In contrast to this MDR pattern, four strains were susceptible to trimethoprim-sulfamethoxazole and the other two isolates were susceptible to chloramphenicol. The second most common PFGE pattern, CN0005, contained 16 isolates, 12 of which showed the MDR pattern of AMP-TCY-CHL-ERY-KAN-NAL-STR-SXT-PB. Other PFGE patterns were represented by fewer than 10 isolates, and 83 of the 101 isolates showed different PFGE patterns, indicating the great genetic heterogeneity of *V. cholerae* O139 isolates in China. Unlike the dominant MDR patterns of antibiotic resistance, the toxigenic O139 strains did not exhibit a dominant genotype associated with PFGE patterns.

### Distribution of the Class I Integron, Super Integron and SXT Element

**Figure 4:**
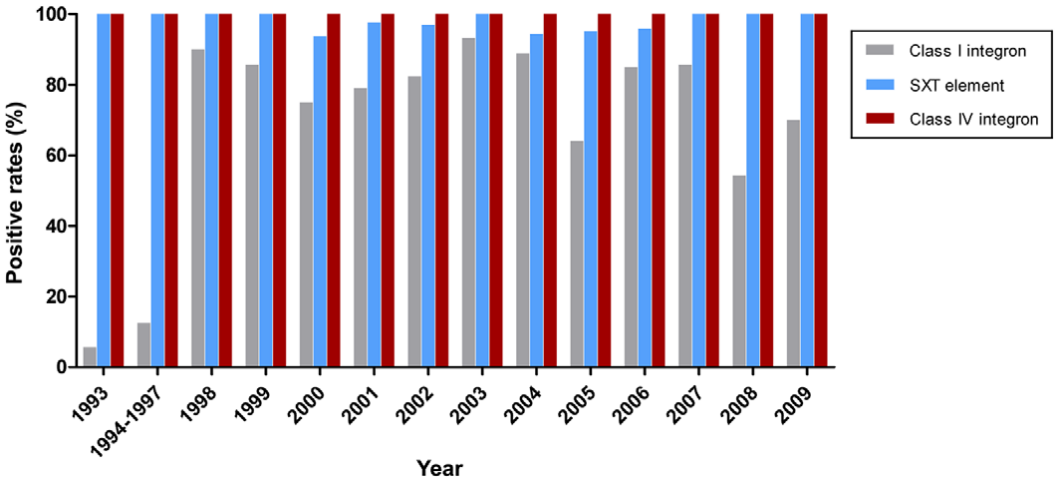
Prevalence of the class I and class IV integrons and the SXT element in toxigenic O139 strains isolated from 1993 to 2009.

The spread of multidrug resistance is facilitated by horizontal transfer via self-transmissible mobile elements [24]. In order to characterize the prevalence of genetic elements associated with multidrug resistance in *V. cholerae* O139 strains, a panel of PCR primers was designed to detect the class I integron, class IV integron and SXT element. The superintegron was identified in all of the O139 isolates by detection of the *int* IV gene. PCR detection of *int* I showed that 71.7% (208) of the toxigenic *V. cholerae* O139 strains were positive for the class I integron, while only 6% (3/50) of the non-toxigenic strains were positive. As shown in Figure 4 and Table S1, the class I integron was first identified in a cholera patient isolate in 1993. More than half of the O139 strains isolated each year since 1998 have been found to contain the class I integron, with rates ranging from 54.3% to 93.3%, indicating significant dissemination of this integron. No obvious increasing trend was identified in the prevalence of the class I integron and year after 1998. PCR assays were designed to detect the variable region of the class I integron and yielded four fragments of different sizes. The class I integron with the typical gene cassette of 1 kb was present in 191 isolates, which contained the *aadA2* gene cassette encoding resistance to streptomycin by sequencing. An amplicon of 1.8 kb was amplified in 15 strains isolated from 1998 to 2009 and were shown to contain *dfrA12*, which encodes the gene for resistance to trimethoprim, *orfF* (unknown function) and *aadA2*. An amplicon of 2.3 kb was detected in only one O139 strain, containing *aar-3*, which encodes resistance to rifampicin, *dfrA27* and *aadA16*, and showed high homology with the sequence of the class I integron in the plasmid pRJ35C of a non-O1/non-O139 strain. The class I integron with gene cassettes of approximately 2.0 kb were detected in four strains that contained the *arr-2* and *aadA3C* genes, which was identical to the sequence of the pIP1202-like plasmid in one of the O139 strains [17].

PCR assays revealed that 97.6% of the toxigenic strains contained the SXT element, while this element was present in only 26.0% of the non-toxigenic strains. As the genes mediating resistance to cholramphenicol, streptomycin and trimethoprim-sulfamethoxazole usually located in the SXT element, we chose these three antibiotic resistances as confounding factors. Stratified analysis revealed significantly higher rates of SXT element in the toxigenic strains than nontoxigenic strains in each stratified group (Supplement Table S2). The SXT element was present in all O139 strains first identified in China in 1993 and the rates of SXT element positive strains has remained at greater than 90.0% from 1993 to 2009. The region containing antibiotic resistance genes in the SXT element was sequenced and analyzed, revealing the presence of the *dfr18*, *floR*, *sulII*, *strA* and *strB* genes among a few isolates. One strain was negative for *dfr18* and all five antibiotic resistance genes were lost in another strain. Both of these deletions have been reported previously [22].

## Discussion

Antibiotics are often used in combination with rehydration therapy. This approach is thought to relieve the symptoms of cholera faster than rehydration treatment alone, in addition to shortening disease duration and reducing the transmission of infectious *V. cholerae* [24]. However, wide usage of antibiotics has increased the sporadic appearance of resistance and multidrug resistant strains are a serious cause for concern.

A number of studies have been reported high prevalence of *V. cholerae* O139 strains resistant to numerous antibiotics recently. In this study, antibiotic resistance patterns were surveyed among *V. cholerae* O139 strains isolated in China from 1993 to 2009 using a panel of 16 antibiotics. Broad MDR was detected especially compared with O1 epidemic strains. A few of the more recently isolated O139 strains isolated were resistant to ciprofloxacin and doxycycline, indicating the careful use of doxycycline as an appropriate antibiotic for treatment of O139 cholera. The spectrum of *V. cholerae* O139 resistance to various antibiotics was narrower at the beginning of its emergence, with a sudden broadening of the resistance of toxigenic *V. cholerae* O139 to various antibiotics in1998, since when the predominant MDR pattern of AMP-TCY-CHL-ERY-KAN-NAL-STR-SXT-PB has spread to large areas of southeastern China, resulting into a severe MDR situation which requires extensive attention. However, the gradual increase in resistance over time was not obvious. Although most strains were sensitive to the new generation of antibiotics, such as ciprofloxacin, doxycycline and azithromycin, the emergence of resistance to these antibiotics may lead to widespread resistance.

The toxigenic O139 strains exhibited significantly higher rates of resistance to nalidixic acid, trimethoprim-sulfamethoxazole, tetracycline, chloramphenicol, azithromycin and kanamycin compared with the non-toxigenic strains. Although the non-toxigenic O139 strains do not cause cholera epidemics and are therefore of less significance to public health, these strains represent clones which toxigenic O139 strains originate from and possess much higher levels of genetic polymorphism [25]. It is speculated that the toxigenic strains are more likely to maintain antibiotic resistance in human infections as a result of the selective pressure of therapeutic treatment, while the non-toxigenic strains are maintained in the natural state in the environment with less antibiotic contact. Furthermore, higher rates of resistance to azithromycin have been detected during some years, thus raising concerns that regarding use of this drug, despite the efficacy of a single dose for treatment of adult cholera patients as reported [21].

The spread of MDR *V. cholerae* O139 strains may be facilitated by the horizontal mobilization of drug resistance genetic elements, such as the class I integron and SXT element [12]. In contrast to *V. cholerae* O1, the initial *V. cholerae* O139 isolates harbored the SXT conjugative element, mediating resistance to streptomycin and trimethoprim-sulfamethoxazole [13]. As previously reported, many O139 strains isolated around the world have acquired the SXT element [13], [22], [26]. Previous studies have also demonstrated that horizontal dissemination of the SXT element is regulated by the bacterial SOS response, which can be induced by ciprofloxacin [27]. This suggests that antibiotics promote the spread of antibiotic resistance genes, resulting in high antibiotic resistance. In this study, the spread of the genetic determinants of resistance, such as the class I integron and the SXT element, were generally synchronized with the trend of increasing antibiotic resistance, indicating a close correlation between genotype and phenotype in *V. cholerae* O139 strains.

An interesting finding of this study was the obvious differences in antibiotic resistance rates and patterns in O139 and O1 strains isolated during the same years, which further suggests separated lineage evolution between these strains. O1 strains are responsible for large-scale outbreaks and prolonged epidemics, whereas O139 strains predominantly cause sporadic cases and foodborne outbreaks in China, such as food poisoning. Studies that compare *V. cholerae* O1 and O139 emphasized the difference of their environmental distribution. It was reported that *V. cholerae* O1 consistently achieved higher abundances than *V. cholerae* O139 in colonizing adults of each copepod species as well as the multiple life stages of *E. affinis* [28]. The difference in colonization may be significant in the general predominance of *V. cholerae* O1 in cholera epidemics in rural Bangladesh where water supplies are taken directly from the environment. Differences of reported cholera cases caused by O1 and O139 may exist in resource utilization and responses to changing environmental conditions in the aquatic habitat [28]. The reason for the significant variance in the antibiotic resistance observed in *V. cholerae* O1 and O139 strains is not clear, although this is partially explained by the fact that the O139 strains exhibited high resistance at its emergence in 1993. The threat of resistance transfer from MDR O139 strains to O1 exists, especially in areas of mixed O139 and O1 cholera epidemics.

It has been speculated that antibiotic therapy is not the principal cause of the generation of MDR in O139 strains based on the observation of predominantly sporadic cases, diverse clones, occasional outbreaks which quickly disappeared in China and relatively low resistance rates of coexistent O1 epidemic strains. Furthermore, no obvious trend of consecutive resistance over time has been observed, in contrast to the complex MDR patterns present in strains isolated in different years. Moreover, with the exception of active transfer of mobile elements carrying resistant genes, the complex clones of serogroup O139 may also contribute to the complexity of antibiotic resistance patterns of O139 strains. It is speculated that the spread of the O139-antigen among different progenitors of *V. cholerae*, combined with genetic rearrangements and natural selection, results in different lineages of O139 strains [12]. Some studies showed continuous genetic changes in *V. cholerae* O139 based on ribotyping and CTX genotyping [5], [6], [8], [9], [12]. Diverse PFGE patterns of O139 isolates were observed in this study. MDR was also found in the environmental non-toxigenic O139 strains in this study, indicating that horizontal transfer of the CTX element may also generate new clones of toxigenic O139 strains with MDR.

In conclusion, the *V. cholerae* O139 strains isolated in China exhibit a broad spectrum of resistance to numerous antibiotics, with multidrug resistant patterns of to five or more antibiotics found to be common in the toxigenic strains. It can be speculated that the high heterogeneity and rapid clonal change of O139 strains results in the rapid shift of MDR patterns. Although O139 cholera is still confined to Asia with no large-scale and prolonged epidemics, the threat to public health posed by MDR O139 strains still exists. Furthermore, the possibility of resistant gene transfer to O1 serogroup and even other species requires a significant concern. Continuous monitoring of the changing trends in antibiotics resistance patterns of O139 serogroup strains may provide the basis of strategies for the control of new cholera pathogens.

## Materials and Methods

### *V. Cholerae* Strains

Using stratified sampling of cases identified in different years from 1993 to 2009, a total of 340 *V. cholerae* O139 strains used in this study were randomly selected from records available at the Centers for Disease Control and Prevention in China. As a Class A infectious disease defined by the Law on the Prevention and Treatment of Infectious Diseases in China, the records included the O139 strains isolated from almost all cholera cases in China. Eighteen *V. cholerae* strains were isolated from the first outbreak in Xinjiang in 1993. A total of 10 strains isolated from the few O139 cholera cases reported from 1994 to1997 were included in this study. Two hundred and fifty-six O139 strains included in this study were obtained from small-scale outbreaks or sporadic cases after 1997 (foodborne outbreaks became common after 1997). A single strain was selected when a group of strains was obtained from one outbreak. In this study, 56 strains were isolated from environmental water and animals (predominantly seafood and turtles) in southeastern China from 1998 to 2009.

### Antibiotic Susceptibility Tests

Antibiotic susceptibility tests were performed using the microbroth dilution method according to the guidelines of the current Clinical and Laboratory Standards Institute (CLSI). Sixteen different antibiotics chosen by antimicrobial mechanism and clinical usage for *V. cholerae* (Sigma, St. Louis, MO,USA) were tested in this study including ampicillin (AMP), cephalothin (CEP), cefuroxime (CXM), ceftriaxone (CRO), cefixime (CFM), tetracycline (TCY), doxycycline (DOX), trimethoprim-sulfamethoxazole (SXT), kanamycin (KAN), streptomycin (STR), nalidixic acid (NAL), ciprofloxacin (CIP), chloramphenicol (CHL), erythromycin (ERY), azithromycin (AZM) and polymyxin B (PB). Multidrug resistance was defined as an absence of susceptibility to two or more classes of antibiotics. *Escherichia coli* ATCC®25922 and *Staphylococcus aureus* ATCC®29213 were used as controls in microbroth dilution antibiotic susceptibility tests and results were analyzed with WHONET 5.4 software (WHO Collaborating Centre for the Surveillance of Antibiotics Resistance, Geneva, Switzerland).

### Molecular Subtyping

Pulsed-field gel electrophoresis (PFGE) is a high-performance molecular subtyping method that has been used for *V. cholerae* previously [29]. Briefly, chromosomal DNA isolated from overnight bacterial cultures was digested overnight with *Not* I at 37°C. The digested fragments were electrophoretically separated using a CHEF-DR III system (Bio-Rad, USA). Electrophoresis conditions were as follows: 6 v/cm at 14°C and pulse times ramped from 2 s to 10 s for 13 hours followed 6 v/cm ramped from 20 s to 25 s for a further 6 h. Images were captured using the Gel Doc 2000 system (Bio-Rad, USA) and the PFGE patterns were analyzed using the BioNumerics software package 4.0 (Applied Maths, Belgium). Using this technique, a subset 177 of 340 *V. cholerae* O139 strains were chosen for analysis of the genetic variation among isolates of different geographic areas.

### PCR and Sequencing of the Class I Integron

The presence of the cholera toxin, class I integron, class IV integron and SXT element was screened by PCR amplification using the primers listed in Table 2. The *int* I primer pair was chosen to amplify the integrase (*int* I) as a marker of the presence of the class I integron. Primer pairs 5′CS-F and 3′CS-R and qacE-F and sul1-R were used to amplify and sequence the variable region and 3′ conserved segment of the integron, respectively. Primer pairs specific for the *int* IV and *int_sxt_*, were used to detect the class IV integron and SXT element respectively. Based on the genes encoding resistance in the SXT element reported previously [13], five primer pairs were used to amplify a product of approximately 17.2 kb in size containing five genes (*dfr18*, *floR*, *sulII*, *strA* and *strB*) in the SXT element.

**Table 2:** Sequences of primers used for detection of cholera toxin and antibiotic resistance genes.

Primer	Sequences (5′–3′)	Amplicon(bp)	GenBankno.
CT-F	ATT TTG AGG TGT TCC ATG TG	749	AAF94613
CT-R	ATA AAG CAG TCA GGT GGT CT		
Int I-F	CGT GTA AAT CAT CGT CGT AG	542	AAF94613
Int I-R	CAA CTG CGG GTC AAG GAT		
5′cs-F	GGC ATCC AAG CAG CAA GC	1913	AF550415
3′cs-R	AAG CAG ACT TGA CCT GAT AG		
qacE-F	TTG CCC CTT CCG CCG TTG TC	997	AF550415
sul1-R	GTG GGT GCG GAC GTA GTC AG		
Int IV-F	AAC ACC GCT TGC ACC TCT AT	525	AF179591
Int IV-R	TGT ATG CGC TTG AGA GTC C		
int_sxt_-F	GCT GGA TAG GTT AAG GGC AG	592	AY055428
int_sxt_-R	CTC TATG GGC ACT GTC CAC ATT		
rumB-F	CGCTCTCGCACTTGTTGTC	3941	AY055428
tnp-R	CAGGGCACGTTGCACATAC		
tnp-F	TACAACGGGATGGCAAGAT	3326	AY055428
drf18-R	GTGGTGTAAACGGTGAGAT		
drf18-F	AAGCGTTTGAGGTCTTCTTT	4721	AY055428
floR-R	CTCAGGACGGTTGGCATAAG		
floR-F	TGTCGCTTTCCGTCTACTT	4009	AY055428
Sul-R	TCTGCCAAACTCGTCGTTAT		
Sul-F	GAACGCCGCAATGTGATCC	3231	AY055428
rumB-R	GAAGGGGCAGAGTTAGAGAT		

## Supporting Information

Table S1 Multidrug resistance patterns and distribution of class I integron and SXT among *V. cholerae* O139 isolated from 1993 to 2009.
(DOC)

Table S2 Stratified analysis of the relationship between SXT element distribution in the toxigenic and non-toxigenic *V. cholerae* O139 strains.
(DOC)
