# Supplementary material for: Multiple Antibiotic Resistance of Vibrio cholerae Serogroup O139 in China from 1993 to 2009
Source: PLoS One. 2012 Jun 11;7(6):e38633. doi: 10.1371/journal.pone.0038633 (PMC3372494; doi:10.1371/journal.pone.0038633)
Supplement: Table S1 — Multidrug resistance patterns and distribution of class I integron and SXT among V. cholerae O139 isolated from 1993 to 2009. (DOC) [file pone.0038633.s001.doc]

**Supplement**

**Table S1.** Multidrug resistance patterns and distribution of class I integron and SXT among *V. cholerae* O139 isolated from 1993 to 2009.

| **MDR** | **No.** | **ctxAB** | **The class I integron** | | **SXT** | **Year** | | | | | | | | | | | | | |
| --- | --- | --- | --- | --- | --- | --- | --- | --- | --- | --- | --- | --- | --- | --- | --- | --- | --- | --- | --- |
|  |  |  | ***int I*** | **gene cassettes** |  | **1993** | **1994-1997** | **1998** | **1999** | **2000** | **2001** | **2002** | **2003** | **2004** | **2005** | **2006** | **2007** | **2008** | **2009** |
| S R | 1 | + | - | - | + |  |  |  | 1 |  |  |  |  |  |  |  |  |  |  |
| PS | 1 | + | - | - | + | 1 |  |  |  |  |  |  |  |  |  |  |  |  |  |
| E S | 1 | - | - | - | - |  |  |  |  |  |  |  |  |  | 1 |  |  |  |  |
| E P | 3 | - | - | - | - |  |  |  | 1 |  |  |  |  |  |  |  |  | 1 | 1 |
| E N | 1 | - | - | - | + |  |  |  |  |  |  |  |  |  |  |  | 1 |  |  |
| PS R | 10 | + | - | - | + | 5 | 3 |  | 1 |  |  |  |  | 1 |  |  |  |  |  |
| E PS | 7 | +(3),-(4) | - | - | +(2),-(5) | 1 |  |  |  | 1 |  |  |  | 2 |  | 2 | 1 |  |  |
| A PS | 1 | - | - | - | + |  |  |  |  |  | 1 |  |  |  |  |  |  |  |  |
| A E S | 2 | - | - | - | - |  |  | 1 |  |  | 1 |  |  |  |  |  |  |  |  |
| E PS R | 19 | +(14),-(5) | - | - | +(15),-(4) | 10 | 3 |  |  | 1 | 2 |  |  |  | 1 | 1 |  |  | 1 |
| E N S R | 1 | - | - | - | + |  |  |  |  |  | 1 |  |  |  |  |  |  |  |  |
| E NPS | 5 | +(4),-(1) | +(1),-(4) | *aadA*2 | +(4),-(1) |  |  |  |  |  | 2 | 1 |  |  |  |  | 1 |  | 1 |
| A E S R | 2 | - | - | - | - |  |  |  |  |  | 1 | 1 |  |  |  |  |  |  |  |
| A E PS | 16 | - | - | - | +(2),-(13) |  | 1 |  |  | 1 | 2 | 1 |  | 1 | 5 | 3 | 1 | 1 |  |
| A E NP | 2 | - | - | - | - |  |  |  |  |  |  |  |  |  |  |  | 1 |  | 1 |
| E NPS R | 4 | + | - | - | + |  | 1 |  |  |  | 1 |  |  |  |  | 2 |  |  |  |
| EK PS R | 1 | + | - | - | + |  | 1 |  |  |  |  |  |  |  |  |  |  |  |  |
| EKNPS | 1 | + | - | - | + |  |  |  |  |  |  |  |  |  | 1 |  |  |  |  |
| A E NPS | 5 | +(2),-(3) | - | - | +(2),-(3) |  |  |  |  |  | 1 |  | 1 |  | 2 | 1 |  |  |  |
| E NPSTR | 7 | + | + | *dfrA12-orfF- aadA2* | + |  |  |  |  |  |  |  | 2 | 2 | 2 |  |  |  | 1 |
| EK PSTR | 7 | + | + | | *aadA2,* | | --- | | *aar-3-dfrA27-aadA16,* | | *arr-2-aadA3C* | | + |  |  |  | 3 | 1 | 2 | 1 |  |  |  |  |  |  |  |
| CE PSTR | 1 | + | - | - | + |  |  |  |  |  |  | 1 |  |  |  |  |  |  |  |
| A KNPS R | 1 | + | + | *aadA*2 | + |  |  |  | 1 |  |  |  |  |  |  |  |  |  |  |
| A E PSTR | 1 | + | + | *aadA*2 | + |  | 1 |  |  |  |  |  |  |  |  |  |  |  |  |
| A E N S R | 2 | + | + | *aadA*2 | + |  |  |  | 1 |  |  | 1 |  |  |  |  |  |  |  |
| AC PSTR | 2 | + | + | *dfrA12-orfF- aadA2* | + |  |  | 2 |  |  |  |  |  |  |  |  |  |  |  |
| EKNPSTR | 19 | + | + | *aadA*2 | + |  |  |  |  |  |  |  | 1 | 1 |  | 3 | 3 | 2 | 9 |
| CE NPSTR | 2 | + | + | *dfrA12-orfF- aadA2* | + |  |  |  |  |  |  |  | 1 |  | 1 |  |  |  |  |
| A EK PSTR | 1 | + | + | *aadA*2 | + |  |  |  | 1 |  |  |  |  |  |  |  |  |  |  |
| A EKNP R | 5 | + | +(2),-(3) | *aadA*2 | + |  |  |  |  |  | 3 |  | 1 |  |  |  |  |  | 1 |
| AC K PSTR | 1 | + | + | *dfrA12-orfF- aadA2* | + |  |  |  | 1 |  |  |  |  |  |  |  |  |  |  |
| ACE PSTR | 2 | + | + | *dfrA12-orfF- aadA2* | + |  |  | 1 | 1 |  |  |  |  |  |  |  |  |  |  |
| ACE NP TR | 1 | + | + | *aadA*2 | + |  |  |  |  |  | 1 |  |  |  |  |  |  |  |  |
| CEKNPSTR | 8 | + | + | *aadA*2 | + | 1 |  |  |  | 1 |  | 3 | 2 |  |  | 1 |  |  |  |
| A EKNPSTR | 17 | + | + | *aadA*2 | + |  |  | 1 |  |  | 1 | 2 | 6 | 3 | 1 | 2 |  |  | 1 |
| ACE NPSTR | 4 | + | + | *aadA*2  *dfrA12-orfF- aadA2* | + |  |  |  |  | 1 | 1 |  |  | 1 | 1 |  |  |  |  |
| ACEK PSTR | 7 | +(2),-(5) | +(3),-(4) | *aadA*2 | +(3),-(4) |  |  |  | 1 | 2 | 1 | 1 | 1 |  | 1 |  |  |  |  |
| ACEKNPST | 16 | +(15),-(1) | +(2),-(14) | *aadA*2 | +(14),-(2) |  |  |  |  |  |  |  |  |  | 11 | 3 |  | 2 |  |
| ACEKNPSTR | 153 | +(151),-(2) | +(132),-(21) | *aadA*2  *dfrA12-orfF- aadA2* | +(151),-(2) |  |  | 7 | 4 | 10 | 29 | 24 | 18 | 9 | 24 | 16 | 6 | 5 | 1 |

A: ampicillin; C: chloramphenicol; E: erythromycin; K: kanamycin; N: nalidixic acid; P: polymyxin B; S: streptomycin; T: tetracycline; R: trimethoprim-sulfamethoxazole.
