# Supplementary material for: Multiple Antibiotic Resistance of Vibrio cholerae Serogroup O139 in China from 1993 to 2009
Source: PLoS One. 2012 Jun 11;7(6):e38633. doi: 10.1371/journal.pone.0038633 (PMC3372494; doi:10.1371/journal.pone.0038633)
Supplement: Table S2 — Stratified analysis of the relationship between SXT element distribution in the toxigenic and non-toxigenic V. cholerae O139 strains. (DOC) [file pone.0038633.s002.doc]

**Supplement**

**Table S2.** Stratified analysis of the relationship between SXT element distribution in the toxigenic and non-toxigenic *V. cholerae* O139 strains.

|  | R3 | |  | R2 | |  | R1 | |
| --- | --- | --- | --- | --- | --- | --- | --- | --- |
|  | CT ^+^ | CT - |  | CT ^+^ | CT - |  | CT ^+^ | CT - |
| SXT ^+^ | 174 | 2 |  | 98 | 7 |  | 11 | 5 |
| SXT - | 3 | 2 |  | 3 | 6 |  | 1 | 28 |
| *χ*^2^ | 33.98 | |  | 29.54 | |  | 16.32 | |
| *p* | <0.01 | |  | <0.01 | |  | <0.01 | |

R3, R2, R1: Resistant to 3, 2, 1 or less antibiotics of chloramphenicol, streptomycin and trimethoprim-sulfamethoxazole respectively. Strains which are sensitive to all 16 antibiotics were merged with R1 group for their much less amount. Pearson *χ*^2^ test was used. CT: Cholera toxin; SXT: the SXT element.
